# Human cardiac stem cells rejuvenated by modulating autophagy with MHY-1685 enhance the therapeutic potential for cardiac repair

**DOI:** 10.1038/s12276-021-00676-x

**Published:** 2021-09-28

**Authors:** Ji Hye Park, Hyeok Kim, Hyung Ryong Moon, Bong-Woo Park, Jae-Hyun Park, Woo-Sup Sim, Jin-Ju Kim, Hye Ji Lim, Yeon-Ju Kim, Seung Taek Ji, Woong Bi Jang, Vinoth Kumar Rethineswaran, Le Thi Hong Van, Ly Thanh Truong Giang, Jisoo Yun, Jong Seong Ha, Kiwon Ban, Hae Young Chung, Sang Hong Baek, Hun-Jun Park, Sang-Mo Kwon

**Affiliations:** 1 Laboratory for Vascular Medicine and Stem Cell Biology, Medical Research Institute, Department of Physiology, School of Medicine, Pusan National University, Yangsan, 50612 Republic of Korea; 2 R&D Center for Advanced Pharmaceuticals & Evaluation, Korea Institute of Toxicology, 141 Gajeong-ro, Yuseong-gu, Daejeon, 34114 Republic of Korea; 3 Department of Medical Life Science, College of Medicine, The Catholic University of Korea, 222 Banpo-Daero, Seocho-Gu, Seoul, 137701 Republic of Korea; 4 Laboratory of Medicinal Chemistry, College of Pharmacy, Pusan National University, Busan, 46241 Republic of Korea; 5 Department of Biomedical Sciences, City University of Hong Kong, Tat Chee Avenue, Kowloon, Hong Kong SAR; 6 Molecular Inflammation Research Center for Aging Intervention, College of Pharmacy, Pusan National University, Busan, 462414 Republic of Korea; 7 Division of Cardiology, Department of Internal Medicine, The Catholic University of Korea, 222 Banpo-Daero, Seocho-Gu, Seoul, 137701 Republic of Korea

**Keywords:** Heart stem cells, Stem-cell therapies

## Abstract

Stem cell-based therapies with clinical applications require millions of cells. Therefore, repeated subculture is essential for cellular expansion, which is often complicated by replicative senescence. Cellular senescence contributes to reduced stem cell regenerative potential as it inhibits stem cell proliferation and differentiation as well as the activation of the senescence-associated secretory phenotype (SASP). In this study, we employed MHY-1685, a novel mammalian target of rapamycin (mTOR) inhibitor, and examined its long-term priming effect on the activities of senile human cardiac stem cells (hCSCs) and the functional benefits of primed hCSCs after transplantation. In vitro experiments showed that the MHY-1685‒primed hCSCs exhibited higher viability in response to oxidative stress and an enhanced proliferation potential compared to that of the unprimed senile hCSCs. Interestingly, priming MHY-1685 enhanced the expression of stemness-related markers in senile hCSCs and provided the differentiation potential of hCSCs into vascular lineages. In vivo experiment with echocardiography showed that transplantation of MHY-1685‒primed hCSCs improved cardiac function than that of the unprimed senile hCSCs at 4 weeks post-MI. In addition, hearts transplanted with MHY-1685-primed hCSCs exhibited significantly lower cardiac fibrosis and higher capillary density than that of the unprimed senile hCSCs. In confocal fluorescence imaging, MHY-1685‒primed hCSCs survived for longer durations than that of the unprimed senile hCSCs and had a higher potential to differentiate into endothelial cells (ECs) within the infarcted hearts. These findings suggest that MHY-1685 can rejuvenate senile hCSCs by modulating autophagy and that as a senescence inhibitor, MHY-1685 can provide opportunities to improve hCSC-based myocardial regeneration.

## Heart disease: keeping cardiac stem cells younger

Stem cells for repairing damaged hearts could be kept in a younger and more viable state using a drug called MHY-1685 which assists processes that resist cell aging. Using stem cells to repair damaged heart tissue requires millions of cells but culturing a suitable cell type often causes many cells to age and become less viable. Ji Hye Park at Pusan University, Yangsan, South Korea, and colleagues explored the potential of MHY-1685 to rejuvenate human cardiac stem cells (hCSCs). The drug enhances a maintenance process called autophagy, which clears cells of worn-out components. Exposure to MHY-1685 generated stem cell populations that proved more effective than unexposed cells at repairing damaged heart tissue when transplanted into rats. Its potential for producing cells for treating patients should be explored.

## Introduction

Myocardial infarction (MI) leads to cardiac functions becoming irreversibly damaged due to the permanent loss of cardiomyocytes (CM) and scar tissue formation^1^. Until now, treatments that prevent additional damage to the ischemic heart or reduce scar formation have been used, but these treatments have not completely prevented the progression of heart failure. Therefore, obtaining effective treatment for cardiac repair is essential. Stem cell-based therapies are promising for acute MI and have been demonstrated to improve cardiac function by functionally forming de novo vessels and CMs and by contributing anti-inflammation effects in a paracrine manner^2^. hCSCs, as a promising cell source, have been examined in animal experimental studies for MI because hCSCs have the potential to differentiate into cardiac cell lineages and promote tissue regeneration in a paracrine/autocrine manner^3–6^. Endogenous or exogenous cardiac restoration mechanisms have been thought to regenerate CMs differentiated from hCSCs^7,8^. However, a study using genetic lineage fate mapping showed that the amount of CMs endogenously regenerated from hCSCs was likely minimal^9^. On the other hand, animal experimental studies showed that the transplantation of in vitro expanded hCSCs could enhance cardiac function, largely through paracrine effects^10,11^.

Although adult stem cells such as hCSCs, mesenchymal stem cells (MSCs), hematopoietic stem cells, and endothelial progenitor cells can be isolated and expanded to transplant autologous cells, open stem cell-based therapy requires a high number of cells to achieve therapeutic benefits^12^. Therefore, repetitive subculture is necessary for expanding the cell numbers to meet the amount required for cell therapy. In addition, repeatedly expanded cells have a problem in that their telomeres gradually shorten with each round of DNA replication, and a critical length that inhibits further replication is eventually reached, causing the cell division to stop and the cell viability to become reduced^13^. This replicative senescence decreases the therapeutic potential of hCSCs not only by activating a senescence-associated secretory phenotype (SASP) but also by inhibiting proliferation and differentiation^14^. Therefore, the discovery of novel function-modulating factors by which hCSCs manage to escape senescence may ultimately help in terms of enhancing the effectiveness and improving the safety of therapeutic strategies.

In general, autophagy is known to play a key role in regulating proliferation and differentiation in a variety of cell types^15^. Conceptually, autophagy and senescence exhibit common characteristics because both phenomena contribute to cellular responses to stress. However, autophagy primarily has an effective anti-senescence role involving various pathways, of which the principal players are the mammalian target of rapamycin (mTOR), adenosine monophosphate-activated protein kinase (AMPK), and p53^16,17^. Recently, Garcia-Prat et al.^17^ reported that basal autophagy was essential to maintain the satellite cell quiescent state and that failure of autophagy might cause the cell to enter into senescence by loss of proteostasis and increased mitochondrial dysfunction and oxidative stress, resulting in a decline in the function and number of satellite cells. Furthermore, Jakovljevic et al.^18^ proposed that the modulation of autophagy in MSCs prior to transplantation enhanced the survival and viability of engrafted MSCs and promoted their proangiogenic and immunomodulatory characteristics. These findings suggest that autophagy is required for maintaining the stemness and enhancing the regeneration potential of stem cells^19^.

In this study, we exploited a novel small molecule, MHY-1685, that possesses antioxidant properties and could inhibit mTOR signaling in a dose-dependent manner and protect against hCSC senescence through autophagy activation. Interestingly, long-term priming of senile hCSCs with MHY-1685 augmented cellular activities with increased cellular proliferation and differentiation, suppressed SASP and the associated inflammation, and led to a significant improvement in cardiac function and remodeling in infarcted hearts. Indeed, this new approach for rejuvenating senile cells can provide a broader perspective on the clinical applications of adult stem cell therapy.

## Materials and methods

### Isolation of the c-Kit-positive hCSCs

Human cardiac tissue was provided by the Pusan National University Hospital. Ethical approval for this study was obtained from the Ethical Review Board of Pusan National University Yang San Hospital, Korea (IRB 05-2015-133), and the present study complied with the Declaration of Helsinki. Informed written consent was given prior to the inclusion of subjects in the study. The heart tissue was minced and incubated in 0.2% collagenase type II (Worthington Biochemical Corp., Lakewood, JU, USA) to disperse the cells. The resulting primary single cardiac cells were expanded to approximately 70‒80% confluency. hCSCs were sorted by magnetic-activated cell sorting using a human c-Kit‒specific antibody.

### Cell culture and characterization of hCSCs

The c-Kit‒positive hCSCs were cultured in F12 Ham’s Medium (HyClone, #SH30026.01, GE Health care, Chicago IL, USA) supplemented with 10% heat-inactivated fetal bovine serum (FBS) (Gibco, Thermo Fisher Scientific, Carlsbad, CA, USA), 1% penicillin/streptomycin (P/S), 2 mM glutathione (Sigma–Aldrich, St. Louis, CA, USA), 10 ng/mL recombinant human basic fibroblast growth factor (Peprotech, Rocky Hill, NJ, USA), and 0.005 unit/mL human erythropoietin (R&D Systems, Minneapolis, MN, USA) (designated hCSC medium). The culture was maintained at 37 °C in a humidified incubator with an atmosphere of 5% CO_2_, and the medium was replaced every 2 days. To characterize hCSCs, cells were probed with human c-Kit, CD29, CD90, CD105, CD44, CD166, CD34, and CD45 antibodies (BD Biosciences, NJ, USA), and the expression of the relevant markers was analyzed employing a BD Accuri flow cytometer (BD Bioscience).

### Chemicals

MHY-1685 was synthesized using a Knoevenagel condensation reaction. A suspension of 4-hydroxybenzaldehyde (300 mg, 2.46 mmol) and barbituric acid (315 mg, 2.46 mmol) in EtOH (4 mL) and H_2_O (4 mL) was heated to 80 °C. Before the reaction temperature reached 80 °C, the reaction mixture became a clear solution. However, a precipitate formed during the reaction time (6 h). After cooling, the solid formed was filtered through a Buchner funnel. The filter cake was washed with water, ethanol, and dichloromethane to produce MHY-1685 (471 mg) as a yellow solid with an 82.6% yield.

5-(4-Hydroxybenzylidene)pyrimidine-2,4,6(1*H*,3*H*,5*H*)-trione (MHY-1685): melting point >300 °C; ^1^H nuclear magnetic resonance (NMR) (400 MHz, DMSO-*d*_6_) *δ* 11.23 (s, 1 H, NH), 11.10 (s, 1 H, NH), 10.79 (s, 1 H, OH), 8.29 (d, 2 H, *J* = 8.8 Hz, 2′-H, 6′-H), 8.17 (s, 1 H, vinylic H), 6.84 (d, 2 H, *J* = 8.8 Hz, 3′-H, 5′-H); ^13^C NMR (100 MHz, DMSO-*d*_6_) *δ* 164.8 (C6), 163.7 (C4′), 163.0 (C4), 156.1 (benzylic C), 150.9 (C2), 139.0 (C2′, C6′), 124.4 (C1′), 116.2 (C3′, C5′), 114.9 (C5); LRMS (ESI-) *m/z* 231 (M-H)^−^.

### Drug treatment

MHY-1685—kindly provided by Prof. Hyung Ryoung Moon (Pusan National University College of Pharmacy)—was diluted in hCSC medium and was used to treat cells at subtoxic concentrations (1 μM).

### Cell viability assay

hCSC viability was determined using a CCK-8 kit (Doingin #DI1701-01, Seoul, South Korea) according to the manufacturer’s instructions. To analyze cell proliferation, 5000 cells/well were seeded in each well of a 96-well plate and incubated for 5 days, and viability was estimated by the CCK-8 kit. Absorbance was measured at 450 nm using a spectrophotometer (TECAN, Grodig, Austria). Each experiment was repeated three times.

### BrdU cell proliferation assay

BrdU-incorporated hCSCs were analyzed using a colorimetric BrdU cell proliferation assay kit (Cell Signaling #6813). Briefly, 5000 cells/well were seeded in each well of a 96-well plate and incubated for 24 h. After 24 h, fresh growth medium supplemented with 1x BrdU was added to the cells and incubated for another 24–48 h. Next, the cells were washed with phosphate-buffered saline (PBS), fixed using a fixing/denaturing solution, and subsequently incubated with a primary antibody for 1 h. Following incubation, the cells were washed with wash buffer and probed with horseradish peroxidase (HRP)-conjugated secondary antibody for 30 min. Following three washes, the cells were incubated with the TMB substrate for 30 min. A stop solution (25 μL, 1 M H_2_SO_4_) was added to each well to stop the reaction. Cell proliferation was measured at a wavelength of 450 nm using a spectrophotometer (TECAN, Grodig, Austria).

### Assessment of the morphological alterations

The cell images were obtained using a light microscope (OLYMPUS, Tokyo, Japan). The cell lengths and widths were measured using ImageJ (free software, https://imagej.nih.gov/ij/).

### Western blotting

Cells were washed with cold PBS and lysed in RIPA buffer (Thermo Fisher Scientific). Protein concentrations were quantified using the BCA protein assay (Thermo Fisher Scientific). Equal amounts of protein were separated on 8‒15% gels using sodium dodecyl sulfate–polyacrylamide gel electrophoresis and transferred onto a polyvinylidene fluoride membrane (Millipore, Billerica, MA, USA). The membrane was blocked for 1 h at room temperature with 5% skim milk diluted in TBS-T. Next, the membrane was probed overnight with primary antibodies against Cyclin E (Santa Cruz #sc-481), CDK2 (Santacruz #sc-748), Cyclin D1 (Santa Cruz #sc-8396), CDK4 (Santa Cruz #sc-56277), GAPDH (Santa Cruz #sc-47724), P16^INK4a^ (Abcam #ab108349), P53 (Abcam #ab32132), P27 (Cell Signaling #2552), mTOR (Cell Signaling #2971), and p-mTOR (Ser2448, Cell Signaling #2532), ATG4A, ATG3) at 4 °C. The membrane was then washed with TBST and incubated with an HRP-conjugated secondary antibody for 1 h at room temperature. The protein bands were visualized using an HRP substrate (Millipore #WBLUR0500) and imaged using an Amersham Imager 600 (GE Healthcare).

### Quantitative reverse transcription-polymerase chain reaction (qRT-PCR)

Total RNA was isolated using TRIzol^®^ (Ambion, Invitrogen, Carlsbad, CA, USA). For qRT-PCR, total RNA was converted into cDNA using the Prime Script 1st Strand cDNA Synthesis Kit (TaKaRa, Shiga, Japan) and oligo (dT) primers. qRT-PCR was performed on a Light Cycler 96 Real-Time PCR System (Roche, Basel, Switzerland) using the FastStart Essential DNA Green Master Mix (Roche, Mannheim, Germany). The expression of each gene was normalized to that of the housekeeping gene GAPDH. The primer sequences used were PECAM (F) 5′-ATTGCAGTGGTTATCATCGGAGTG-3′, (R) 5′-CTCGTTGTTGGAGTTCAGAAGTGG-3′;^20^ ACTA2 (F) 5′-AGCAGGCCAAGGGGCTATATAA-3′, (R) 5′-CGTAGCTGTCTTTTTGTCCCATT-3′;^21^ CNN1 (F) 5′-AGGCTCCGTGAAGAAGATCA-3′, (R) 5′-CCACGTTCACCTTGTTTCCT-3′; and GATA6 (F) 5′-ACTCGGGTTGTGTAGGATGC-3′, (R) 5′-GTGCTGGTGAACCTTTTGGT-3′.

### Xenograft model

Six- to eight-week-old male BALB/c nude mice were purchased from Orient Bio (Seoul, Korea). The animal experiments were performed with the approval of the Pusan National University Institutional Animal Care and Use Committee in accordance with the approved guidelines. A total of 1 × 10^7^ hCSCs and colorectal cancer cell lines (DLD-1 and HCT-8) were resuspended in 100 µl of PBS (Matrigel 1:1 mix) and subcutaneously injected into the left and right flanks of the mice. The tumor length and width were measured by calipers at 2 weeks after transplantation. Tumor volumes were calculated as [width × width × length]/2.

### Tube formation assay

A tube formation assay was performed to assess the ability of hCSCs to form tubes. hCSCs (8000 cells) were seeded in a 96-well plate (Thermo Fisher Scientific) coated with growth factor-reduced Matrigel (55 μL/well, BD Biosciences, San Diego, CA) and cultured at 37 °C in an atmosphere containing 5% CO_2_ for over 6 h. Subsequently, the growth of capillary-like structures was monitored by phase-contrast microscopy, images were captured, and the total number of tubes was counted and quantified.

### Senescence-associated beta-galactosidase (SA β-gal) assay

Drug-induced hCSC senescence was investigated by quantifying the number of cells expressing senescence-associated β-galactosidase using a senescence-associated β-galactosidase staining kit (#9860, Cell Signaling Technology, Beverly, MA, USA) according to the manufacturer’s instructions. After staining, images were captured using a light microscope (OLYMPUS, Tokyo, JAPAN).

### Cell death assay

hCSCs were preconditioned with MHY-1685 for 24 h in low serum (2% FBS) Ham’s F12 medium. After incubation, hCSCs were washed with PBS and treated for 4 h with 1 mM H_2_O_2_ (Sigma-Aldrich) diluted in a low serum medium. The cells were harvested and resuspended in 1× annexin binding buffer supplemented with Annexin V (BD Pharminogen, #559763, San Diego, CA, USA) and 7-amino-actinomycin D (7-AAD).

### Differentiation assay

To evaluate the ability of hCSCs to differentiate into ECs, hCSCs were cultured in a DMEM high glucose medium supplemented with 20% FBS, 1% P/S, and 30 ng/mL recombinant human basic fibroblast growth factor (R&D Systems) for 7 days. To differentiate hCSCs into smooth muscle cells (SMCs), hCSCs were cultured in Medium 231 supplemented with 5% FBS, 1% P/S, and 1× smooth muscle differentiation supplement (Gibco) for 7 days. The medium was changed every 2 days. To induce cellular differentiation into CM, hCSCs were cultured in a MEM/EBSS medium (HyClone) supplemented with 2% FBS, 1% P/S, and 10 nM dexamethasone (Sigma-Aldrich) for 28 days. The medium was changed every day. The ability of cells to differentiate was analyzed by qRT-PCR and immunofluorescence.

### Immunofluorescence

For immunofluorescence experiments, hCSCs were fixed with 4% paraformaldehyde for 10 min and permeabilized using 0.25% PBST, and nonspecific binding sites were blocked for 1 h using 5% normal goat serum prepared in PBST. Next, the cells were probed overnight with LC3 (Abcam, 1:200), LAMP1 (Abcam, 1:200), α-SA (Sigma, 1:200), and α-SMA (Sigma-Aldrich, 1:200) antibodies at 4 °C. The cells were then incubated with Alexa Fluor 488 (α-SMA), 555 (LC3), or 647 (LAMP1) secondary antibody (Invitrogen) and 4′,6-diamidino-2-phenylindole (DAPI, Sigma-Aldrich) to stain the nuclei. Images were visualized using a confocal microscope (Olympus, FV-2000, Tokyo, Japan) and Lionheart FX automated microscope (BioTek, Winooski, VT, USA).

### Measurement of the intracellular ROS levels

For the H2-DFFDA (5-(and- 6-) carboxy-2′,7′-dichlorodihydrofluorescein diacetate) assay, hCSCs were harvested using Accutase (Sigma-Aldrich, St. Louis, CA, USA) and incubated with 10 μM carboxy-H2-DFFDA at 37 °C in the dark. After washing with PBS, the cells were resuspended in 100 μL of FACS buffer (2% FBS prepared in 0.2 mM PBST).

### RNA sequencing

Total RNA (2 μg) was used to purify and fragment the mRNA samples. The RNA-seq library preparation was performed by the Teragen Bio Institute (Suwon, South Korea). The differentially expressed gene (DEG) analysis was performed based on the Cuffdiff method. DEG analysis was based on a *q*-value threshold < 0.05 to correct the errors introduced by multiple testing. DEGs were classified using the Gene Ontology (GO) database, which categorizes genes into three main categories, i.e., biological process, cellular component, and molecular function.

### Autophagy assay

hCSCs were seeded in 96-well microplates and incubated overnight. After incubation, the cells were treated with various concentrations of MHY-1685 and 7.5 µM chloroquine (CQ). After overnight incubation, the medium was removed, and the cells were carefully washed with 1× assay buffer. The cells were stained with a CYTO-ID autophagy detection kit (Enzo Linfe Science, Farmingdale, NY, USA) according to the manufacturer’s protocol. Rapamycin was used a positive control. The stained cells were analyzed on a fluorescence microplate reader (SpectraMAX M2e, Molecular Devices, San Jose, CA, USA). For immunofluorescence analysis, hCSCs were treated with a combination of 1 µM MHY-1685 and 10 µM chloroquine (Sigma Aldrich). After 24 h of incubation, the cells were fixed and stained with LC3 and LAMP1 antibodies for autophagic puncta detection.

### DiI (1,1′-dioctadecyl-3,3,3′,3′-tetramethylindocarbocyanine perchlorate) labeling

hCSCs were labeled for tracking with CellTracker^TM^ CM-DiI (Invitrogen #C7000). For the labeling process, we suspended the cells at 1 × 10^6^ cells/mL in DPBS and added DiI at 1 µM/mL. After incubation for 5 min at 37 °C and then 15 min at 37 °C, the cells were centrifuged at 1500 rpm for 5 min and washed twice with DPBS. To prepare cells for transplantation, DiI-labeled hCSCs (1 × 10^6^) were suspended in 100 μL of saline.

### Transplantation of hCSCs in a rat MI model

All animal studies were approved by the Institutional Animal Care and Use Committee (IACUC) of The Catholic University of Korea (Approval number: CUMC-2020-0051-01). IACUC and the Department of Laboratory Animals (DOLA) at The Catholic University of Korea, Songeui Campus accredited the Korea Excellence Animal Laboratory Facility from Korea Food and Drug Administration in 2017 and acquired full AAALAC International accreditation in 2018. All animal procedures conformed to the guidelines from Directive 2010/63/EU of the European Parliament on the protection of animals used for scientific purposes or the NIH guidelines. Male Fischer 344 rats (F344, 8 weeks old, 160–180 g) procured from Koatec, Korea, were anesthetized via inhalation of 2% isoflurane and intubated through the trachea with an 18 G intravenous catheter. The rats were then mechanically ventilated with medical-grade oxygen. A heating pad at 37 °C was used to maintain the body temperature of the rats throughout the operation. The chest was shaved and sterilized with 70% alcohol. Next, a left thoracotomy was performed, and MI was induced by permanent ligation of the left anterior descending artery. The cells were transplanted immediately after the induction of MI; senescent hCSCs (1 × 10^6^ cells per rat) and hCSCs pretreated with MHY-1685 (1 × 10^6^ cells per rat) were intramyocardially injected at two different sites at the border zone. All rats received the following immunosuppressants daily: azathioprine, 2 mg/kg; cyclosporine A, 5 mg/kg; and methylprednisolone, 5 mg/kg. After 4 weeks, the animals were euthanized to perform histology studies.

### TUNEL assay

A terminal deoxynucleotidyl-transferase-mediated dUTP nick end-labeling (TUNEL) kit (Roche #11684795910) assay was used to identify cellular apoptosis. The sections were deparaffinized with xylene and rehydrated. The sections were permeabilized with 200 μl of TBS-T for 2 min on ice. The sections were incubated in a staining solution containing terminal deoxynucleotidyltransferase (TdT) for 1 h at 37 °C in the dark, according to the manufacturer’s protocol. After washing with PBS, the sections were mounted with DAPI mounting solution (Vector; H-1500).

### Echocardiography

Echocardiography was performed to confirm the improvement of cardiac function after treatment. The animals were anesthetized with 2% isoflurane and placed on a heating pad to maintain body temperatures of 37 °C. The chests of the animals were shaved, and ultrasound gel was applied. Echocardiographic images were obtained using an HP SONOS 7500 ultrasound system equipped with an L12-5 linear broadband and a 52 Hz L15-7io linear transducer (Affniti 50 G, Philips). Echocardiography was performed at 1–4 weeks after treatment, and the following measurements were taken: ejection fraction (EF), fractional shortening (FS), left ventricular end-diastolic diameter (LVIDd), left ventricular end-systolic diameter (LVIDs), septal wall thickness (SWT), and posterior wall thickness (PWT). To eliminate bias, a technician performed the echocardiogram in a blinded manner.

### Immunohistochemistry

The hearts were excised at 7 and 28 days following hCSC transplantation and fixed with 4% paraformaldehyde. The tissues were then embedded in a paraffin block and sectioned into 4 μm slices. The sections were deparaffinized, and antigen retrieval was performed using a retrieval solution (DAKO, K8005). Subsequently, the sections were permeabilized in PBS containing 0.5% Triton X-100 for 15 min and then probed at 4 °C with primary antibodies diluted in an antibody diluent containing background reducing components (DAKO, S3022). The following primary antibodies were used in this study: cTnT (Abcam, 1:200), CHP (3Helix, 1:200), CD31 (R&D, 1:200), Ki-67 (Abcam, 1:200), and IL-B4 (Vector, 1:40). Next, after washing three times with PBS, the sections were incubated with fluorescent secondary antibodies (anti-mouse IgG Alexa Fluor 488, anti-goat IgG Alexa Fluor 488, and anti-rabbit IgG Alexa Fluor 647) for 120 min at room temperature in the dark. After washing three times with PBS, the sections were counterstained with DAPI (Vecta Shield) for nuclear staining and then mounted on slides. The heart sections were imaged using a fluorescence microscope (Nikon Eclipse TS2) and confocal microscope (Zeiss, and Lionheart FX automated microscope (Biotek, USA)).

### Determination of fibrosis

MT staining (Sigma, St. Louis, MO, USA) was performed to determine the fibrotic region of the MI hearts. Briefly, three frozen sections were fixed in Bouin’s solution at 56 °C for 15 min in each group. These sections were then stained using Weigert’s iron hematoxylin solution for 5 min at room temperature and then stained with Biebrich scarlet-acid fuchsin solution for 2 min at room temperature. Finally, the sections were counterstained with aniline blue for 5 min, which was followed by incubation in 1% acetic acid for 2 min at room temperature. Extensive washes were performed between each step. The collagen fibers appeared blue, and the viable myocardium appeared red. The percent of the area of fibrosis to the area of the entire left ventricular wall was quantified using ImageJ with basic add-ons.

### Capillary density measurement

At the time of euthanization, the hearts were fixed in 4% paraformaldehyde overnight, and blocks were prepared. After being stored at −20 °C, the hearts were sectioned into 4 μm sections each, starting from the top to the apex, using a microtome (Leica, RM2255, Germany). The number of capillaries was counted by CD31 staining and in five random microscopic fields using a fluorescence microscope (Nikon) and was expressed as the number of capillaries per square mm tissue area.

### Data analysis

All quantitative data are shown as means ± S.E.M. unless otherwise indicated. Statistical differences between two groups were analyzed using a two-tailed Student’s *t*-test. Significant differences between 3 or more groups were also analyzed by ANOVA with Bonferroni’s post hoc analysis. The results were considered significant when the *p* value was less than 0.05.

## Results

### Priming MHY-1685 provides a cytoprotective effect against oxidative stress

First, we found a novel anti-senescence drug called MHY-1685 (Supplementary Fig. 1a) by performing a drug screening that assessed SMP 30 expression in senescent hCSCs as an anti-senescence marker (Supplementary Fig. 1b). Initially, to identify the concentration of MHY-1685 suitable for treatment, a cytotoxicity assay was performed after 24 h of treatment with various concentrations of MHY-1685. The cells were found to be relatively tolerant to concentrations of up to 10 μM (Supplementary Fig. 1c); hence, this concentration was chosen to be used in subsequent experiments. To investigate whether MHY-1685 affected H_2_O_2_-induced ROS generation in hCSCs, we performed H_2_-DFFDA and MitoSOX assays following an MHY-1685 pretreatment of hCSCs for 24 h before incubation with 600 μM H_2_O_2_ for 1 h. Short-term treatment of hCSCs with MHY-1685 significantly reduced intracellular ROS (Supplementary Fig. 1d, e) and mitochondrial superoxide production (Supplementary Fig. 1f) under conditions of H_2_O_2_-induced oxidative stress. Excessive ROS production leads to apoptosis, but hCSCs primed with MHY-1685 showed a significantly reduced number of apoptotic and necrotic cells compared to that of the vehicle control. These results indicate that MHY-1685 exhibits cytoprotective effects against oxidative stress.

### Priming MHY-1685 increases the proliferative potential of hCSCs

**Fig. 1:**
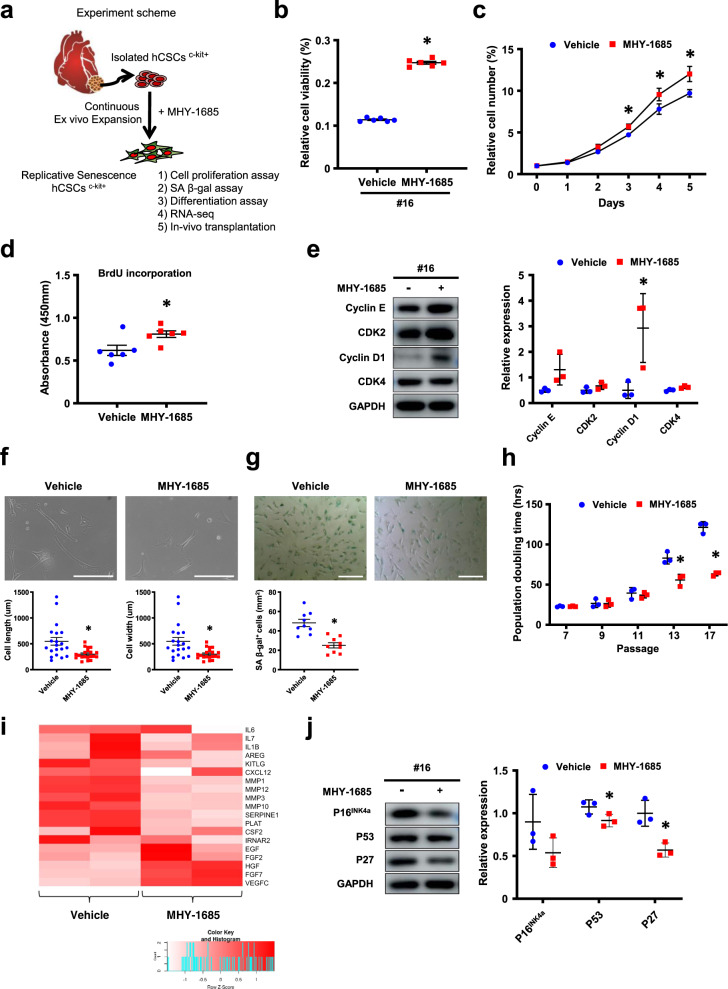
MHY-1685 attenuated the hCSC senescent phenotype. **a** Schematic diagram of the experimental design. **b** After a long-term culture with vehicle or MHY-1685, the relative percentage of cell viability was measured by a CCK-8 assay (*p < 0.05 vs. vehicle). **c** hCSCs were continuously treated with vehicle or MHY-1685, and the proliferation rate of hCSCs was measured by a CCK-8 assay (*p < 0.05 vs. vehicle). **d** After treatment with vehicle or MHY-1685, the S-phase cells were quantified by a BrdU incorporation assay (*p < 0.05 vs. vehicle). **e** Expression of the cell cycle-related proteins Cyclin E, CDK2, Cyclin D1, and CDK4 as quantified by western blot assay (*p < 0.05 vs. vehicle). **f** Representative images of the cell morphology of hCSCs at the same passage. The cell length and width were measured and are presented as a graph (*p < 0.05 vs. vehicle). Scale bar: 100 µm. **g** After long-term culture with vehicle or MHY-1685, cellular senescence was quantified by SA-β-gal-positive cells (*p < 0.05 vs. vehicle). Scale bar: 50 µm. **h** Population doubling time of hCSCs with vehicle or MHY-1685 (*p < 0.05 vs. vehicle). **i** Heat map analysis of SASP-related genes after treatment with vehicle or MHY-1685. **j** Expression of P16^INK4a^, p53, and p27 as quantified by a western blot assay (*p < 0.05 vs. vehicle). Data are shown as the mean ± S.E.M.

To determine whether long-term priming of MHY-1685 affected the proliferative capacity of senescent hCSCs, cells were cultured and expanded in the presence or absence of MHY-1685 (1 μM) (Fig. 1a). At passage 16, in which hCSCs usually become senescent, the MHY-1685‒primed hCSC group showed increased viability compared to that of the vehicle group (Fig. 1b). Cell proliferation using the CCK-8 assay revealed that the MHY-1685-primed hCSC group had a higher proliferation rate (Fig. 1c) with BrdU incorporation (Fig. 1d) than the vehicle group. The expression of Cyclin D1 was significantly increased in hCSCs primed with MHY-1685 compared with that of the vehicle group (Fig. 1e). These data suggest that continuous exposure to MHY-1685 promotes the proliferative ability of senescent hCSCs. Cyclin D1, a cell cycle regulator, is an important protein for cell growth and is well known as a proto-oncogene^22^. In our experiment, long-term treatment of hCSCs with MHY-1685 resulted in cyclin D1 that was overexpressed compared to that of senescent hCSCs. To investigate whether MHY-1685-treated hCSCs have oncogenic effects, we conducted tumorigenesis assays in immune-deficient nude mice. A total of 1 × 10^7^ cells of hCSCs, MHY-1685-treated hCSCs, DLD-1 cells, and HCT-1 cells were subcutaneously injected into the left and right flank regions. DLD-1 and HCT-1 are colorectal cancer cell lines and were used as positive controls to induce tumorigenesis. After 2 weeks, tumors occurred in the groups that were injected with colorectal cancer cell lines (DLD-1, HCT-8); however, tumors were not detected in the only vehicle (PBS) group or, the hCSC- and MHY-1685-primed hCSC group. This result suggests that treating hCSCs with MHY-1685 in the long term did not cause tumors, although the cell cycle of hCSCs treated with MHY-1685 was upregulated (Supplementary Fig. 2).

### Priming MHY-1685 attenuates the senescent phenotype of hCSCs

To investigate whether continuous exposure to MHY-1685 attenuated the senescent phenotype of hCSCs, we examined senescence-associated morphological changes after repeated ex vivo culture. When cultured in the presence of MHY-1685 (1 μM), hCSCs displayed significantly reduced size without a flattened appearance compared with those cultured in the absence of MHY-1685 (Fig. 1f). Next, we measured SA β-gal activity and observed that continuous exposure to MHY-1685 significantly reduced the number of β-gal-positive cells (Fig. 1g). Because the doubling time of cells was continuously increased, which meant the cells had reduced proliferative abilities as cellular aging progressed, hCSCs were continuously subcultured in the presence of vehicle or MHY-1685. Continuously culturing hCSCs in the presence of MHY-1685 significantly decreased doubling time (Fig. 1h). Heat map analysis of RNA-seq data revealed that the expression of SASP-related genes was down-regulated when hCSCs were cultured in the presence of MHY-1685 (Fig. 1i). Furthermore, the protein levels of p16^INK4a^, p53, and p27 were also reduced in the presence of MHY-1685 (Fig. 1j). Taken together, these results suggest that MHY-1685 rejuvenates senescent hCSCs by attenuating the senescent phenotype.

### Priming MHY-1685 improves the differentiation potential of hCSCs

**Fig. 2:**
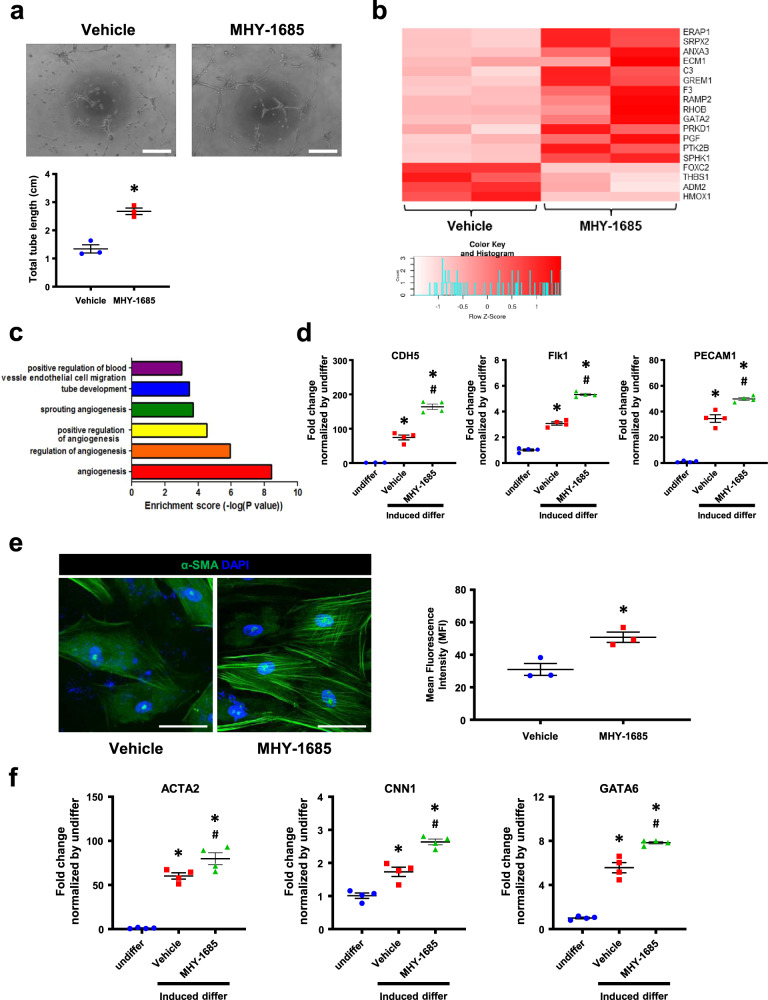
MHY-1685 improved the differentiation capacity of senescent hCSC. **a** Endothelial tube formation ability of senescent hCSCs was determined by a tube formation assay (*p < 0.05 vs. vehicle). Scale bar: 50 µm. **b** Heatmap analysis of the positive regulation of gene expression related to angiogenesis. **c** Enrichment of angiogenesis-related GO terms of genes that were significantly regulated in the MHY-1685-primed hCSCs. **d** Expression of CDH5, Flk1, and PECAM1 after differentiation into endothelial cell lineage as examined by qRT-PCR (*p < 0.05 vs. undifferentiated hCSCs, ^#^*p* < 0.05 vs. vehicle). **e** Vascular smooth muscle cell differentiation ability as evaluated by fluorescent immunocytochemistry using the myocyte marker α-SMA. α-SMA (green), DAPI (blue). (*p < 0.05 vs. vehicle). Scale bar: 50 μm. **f** Expression of ACTA2, CNN1, and GATA6 after differentiation into the vascular smooth muscle cell lineage as examined by qRT-PCR (*p < 0.05 vs. undifferentiated hCSCs, ^#^*p* < 0.05 vs. vehicle). Data are shown as the mean ± S.E.M.

Since hCSCs are able to differentiate cardiac lineage cell types (EC, SMC, and CM), we evaluated the differentiation ability of hCSCs continuously treated with MHY-1685 in three cell types. First, to investigate whether MHY-1685 affected the EC differentiation of hCSCs, we induced hCSC EC differentiation and performed a Matrigel tube formation assay. The total tube length was significantly increased in the MHY-1685-primed hCSC group compared with the vehicle group (Fig. 2a). Heat map analysis revealed that genes related to proteins that promote angiogenesis, such as *SRPX2*, which regulates EC migration and tube formation; PGF, a member of vascular endothelial growth factor subfamily and a key molecule in angiogenesis; and GREM1, a putative angiogenesis-modulating gene, were upregulated in the MHY-1685-primed hCSC group compared to the vehicle group (Fig. 2b). Enrichment of angiogenesis-related GO terms showed that these genes were significantly regulated in the MHY-1685‒primed hCSC group (Fig. 2c). In addition, the transcription of *CDH5*, *Flk1*, and *PECAM1* was significantly increased in the MHY-1685-primed hCSC group compared to that in the other groups (Fig. 2d). These results suggest that MHY-1685 improves the potential of hCSC differentiation into ECs.

To investigate whether MHY-1685 also affected the vascular SMC differentiation of hCSCs, we induced smooth muscle hCSC differentiation and used immunocytochemistry to verify this process. Staining with smooth muscle α-actin, the smooth muscle marker, revealed that MHY-1685 also enhanced the differentiation of hCSCs into vascular SMCs compared to the vehicle group (Fig. 2e). Concordantly, the transcription of *ACTA2, CNN1*, and *GATA6* was also significantly increased in the MHY-1685-primed hCSC group compared to that in the other groups (Fig. 2f). Although enrichment of muscle development-/proliferation-related GO terms showed that these genes were significantly regulated in the MHY-1685-primed hCSC group (Supplementary Fig. 3a), myogenic differentiation was not significantly affected by MHY-1685 treatment compared to the vehicle group (Supplementary Fig. 3b). Consequently, these results indicate that MHY-1685 promoted the differentiation properties of senescent hCSCs.

### Priming MHY-1685 regulates mTOR signaling and the associated autophagy in hCSCs

**Fig. 3:**
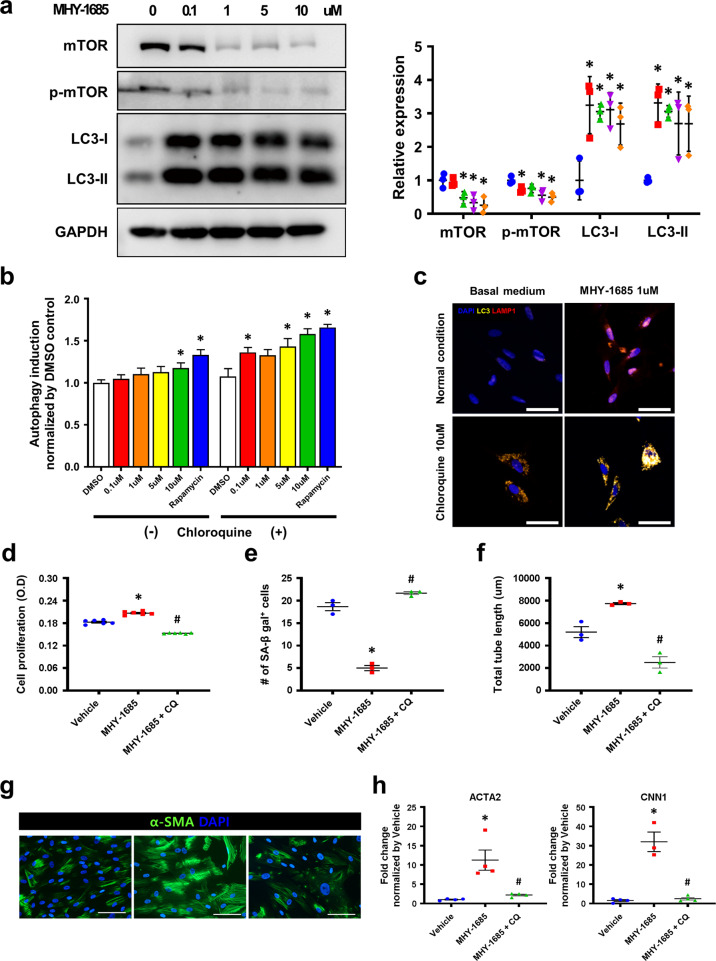
MHY-1685 regulates mTOR and the associated autophagy in hCSCs. **a** Protein expression of autophagy markers mTOR, p-mTOR, LC3-I, LC3-II as evaluated by western blotting (*p < 0.05 vs. vehicle). **b** Autophagy detection with various concentrations of MHY-1685 in senescent hCSCs by CYTO-ID (*p < 0.05 vs. vehicle). **c** hCSCs were immunostained with anti-LC3 antibody (yellow), anti-LAMP1 (red), and DAPI (blue) to identify the occurrence of autophagy after treatment with MHY-1685. Scale bar: 50 μm. **d** The proliferative ability of senescent hCSCs (vehicle, 1 µM MHY-1685-treated hCSCs, 1 µM MHY-1685 + 20 µM + CQ-treated hCSCs) measured by a CCK-8 assay after treatment with MHY-1685 + CQ (*p < 0.05 vs. vehicle, ^#^*p* < 0.05 vs. MHY-1685) **e** The senescence of senescent hCSCs was verified by counting the SA-β-gal-positive cells after treatment with MHY-1685 + CQ (*p < 0.05 vs. vehicle, ^#^p < 0.05 vs. MHY-1685). **f** The differential ability of senescent hCSCs into ECs was verified by a tube formation assay after treatment with MHY-1685 + CQ (*p < 0.05 vs. vehicle, ^#^*p* < 0.05 vs. MHY-1685). **g** Representative image of the differential ability of senescent hCSCs to differentiate into SMCs after treatment with MHY-1685 + CQ. α-SMA (green), DAPI (blue). Scale bar: 100 µm. **h** The differential ability of senescent hCSCs into SMCs was evaluated by qRT-PCR (*p < 0.05 vs. vehicle, ^#^*p* < 0.05 vs. MHY-1685). Data are shown as the mean ± S.E.M.

Recent studies have reported that autophagy plays a role in the modulation of cell proliferation, differentiation, and senescence. Our previous study suggested that the autophagy inducer rapamycin reduced the senescence of hCSCs and improved cellular activities^14^. To establish whether MHY-1685 modulates autophagy signaling, we assessed the expression of an autophagy signal regulator protein (mTOR) by western blotting. We observed that MHY-1685 exhibited a dose-dependent inhibition of mTOR. Moreover, MHY-1685 directly inhibited mTOR signaling and the activation of LC3-I and LC3-II, which are downstream signals of autophagy (Fig. 3a). Subsequently, we performed an autophagy detection assay to analyze the regulation of autophagy by MHY-1685. The autophagy detection assays revealed that MHY-1685 increased autophagy in a dose-dependent manner under basal conditions (Fig. 3b). However, since autophagic vesicles can be rapidly degraded by lysosomes under basal conditions, the regulation of autophagic flux by MHY-1685 may not be accurately analyzed. Chloroquine (CQ) is mainly used as a useful tool in assessing autophagic flux by inhibiting fusion between autophagosomes and lysosomes, which is the final step of autophagy. Thus, we used CQ, which accumulates autophagic vesicles by inhibiting the formation of autolysosomes, to accurately detect the induction of autophagy by MHY-1685 (Fig. 3b). Subsequently, we investigated the increase in autophagy signaling and autophagic flux induced by MHY-1685 by examining the immunocytochemistry of LC3 (a marker of autophagosomes) and LAMP1 (a marker of lysosomes). As expected, the immunocytochemistry results showed that treatment with MHY-1685 increased the number of autophagic puncta (Fig. 3c). Taken together, these results show that MHY-1685 stimulates the autophagy signal of senescent hCSCs.

To investigate the effect of autophagy inhibition on the cellular activity of MHY-1685-primed hCSCs, we analyzed the proliferative ability and senescence phenotype of hCSCs continuously treated with MHY-1685 and CQ. The proliferation rate of senescent hCSCs was significantly deceased when MHY-1685 and CQ were simultaneously treated (Fig. 3d). Furthermore, an SA-β assay revealed that the inhibition of autophagy in MHY-1685-primed hCSCs accelerated the senescence phenotype, suggesting that the cellular activity of MHY-1685-primed hCSCs was improved by regulating the autophagy mechanism (Fig. 3e). Next, we investigated the differential ability of MHY-1685-primed hCSCs after autophagy inhibition. Tube formation assays revealed that the differential ability of hCSCs into ECs was significantly decreased by inhibiting autophagy (Fig. 3f). Subsequently, the differential ability of hCSCs to differentiate into SMCs was verified by α-SMA staining, and the gene expression of *ACTA2* and *CNN1* was also significantly decreased by the inhibition of autophagy signaling (Fig. 3g, h). Taken together, these results show that the senescent hCSCs continuously treated with MHY-1685 were rejuvenated and regained differential ability through regulating the autophagy mechanism.

### Priming MHY-1685 enhances the survival and engraftment of hCSCs in vivo

**Fig. 4:**
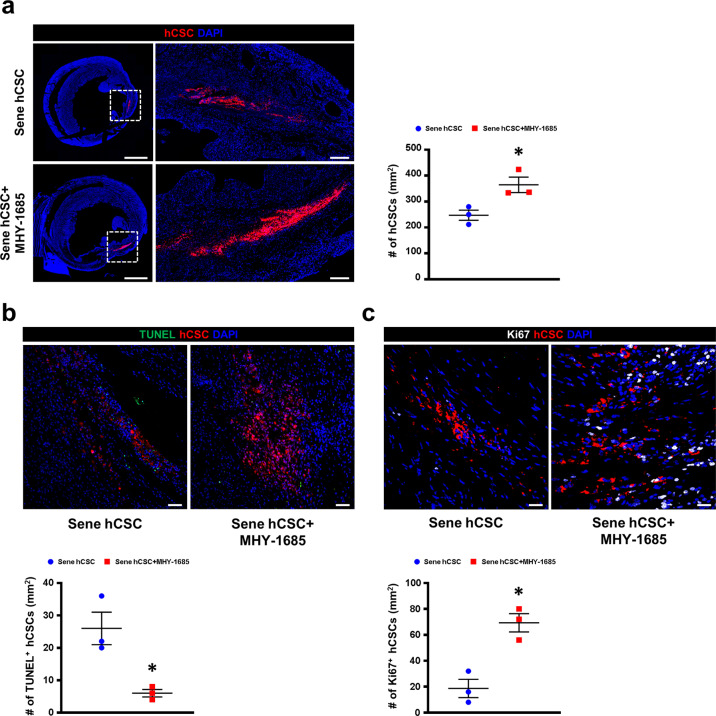
hCSC-primed MHY-1685 enhances cell survival after hCSC transplantation. **a** Representative images of hCSC retention at 1 week after hCSC transplantation and their quantification summary. hCSCs (white), DAPI (blue). *n* = 3. (*p < 0.05 vs. senescent hCSC). Scale bar: 2000 µm (Left), 200 µm (Right). **b** Representative images of the TUNEL assay in the infarct zone 1 week after MI and their quantification summary. TUNEL (green), hCSC (red), DAPI (blue). *n* = 3. (*p < 0.05 vs. senescent hCSC). Scale bar: 50 µm. **c** Representative images of proliferative hCSCs stained with Ki-67 in the infarct zone 1 week after MI and their quantification summary. Ki-67 (white), hCSC (red), DAPI (blue). *n* = 3. (*p < 0.05 vs. senescent hCSC). Scale bar: 20 µm. Data are shown as the mean ± S.E.M.

To establish the therapeutic efficacy of hCSCs, senescent hCSCs cultured in a medium containing either DMSO (Sene hCSC group) or MHY-1685 (Sene hCSC+MHY-1685 group) were transplanted into the rat MI hearts. After permanent ligation of the left anterior descending (LAD) artery, 1 million hCSCs (stained with Dil for cell retention and tracking) were injected into the border zone of the left ventricular myocardium. Since the therapeutic effect of stem cell-based therapy for cardiac regeneration depends on the survival and engraftment of transplanted cells in the myocardium after transplantation, we first investigated the retention of hCSCs in the infarcted area one week following MI (Fig. 4a, Supplementary Fig. 4). The retention of hCSCs was significantly higher in the MHY-1685‒primed hCSC group than in the senescent hCSC group (Fig. 4a, Supplementary Fig. 4). Subsequently, we performed histological assessment using TUNEL and Ki-67 staining to investigate why the retention of hCSCs differed between the senescent hCSC group and the MHY-1685-primed hCSC group. Apoptotic hCSCs, which were double-positive for TUNEL and Dil, were significantly decreased in the MHY-1685-primed hCSC group compared to the senescent hCSC group (Fig. 4b). Proliferative hCSCs, which were double-positive for Ki-67 and DiI, were significantly improved in the MHY-1685-primed hCSC group compared to the senescent hCSC group (Fig. 4c). These data suggest that MHY-1685-primed hCSCs exhibited enhanced cell engraftment and survival, which reduced apoptosis and increased proliferation, as confirmed in vitro.

### hCSC-primed MHY-1685 improves cardiac function in MI

**Fig. 5:**
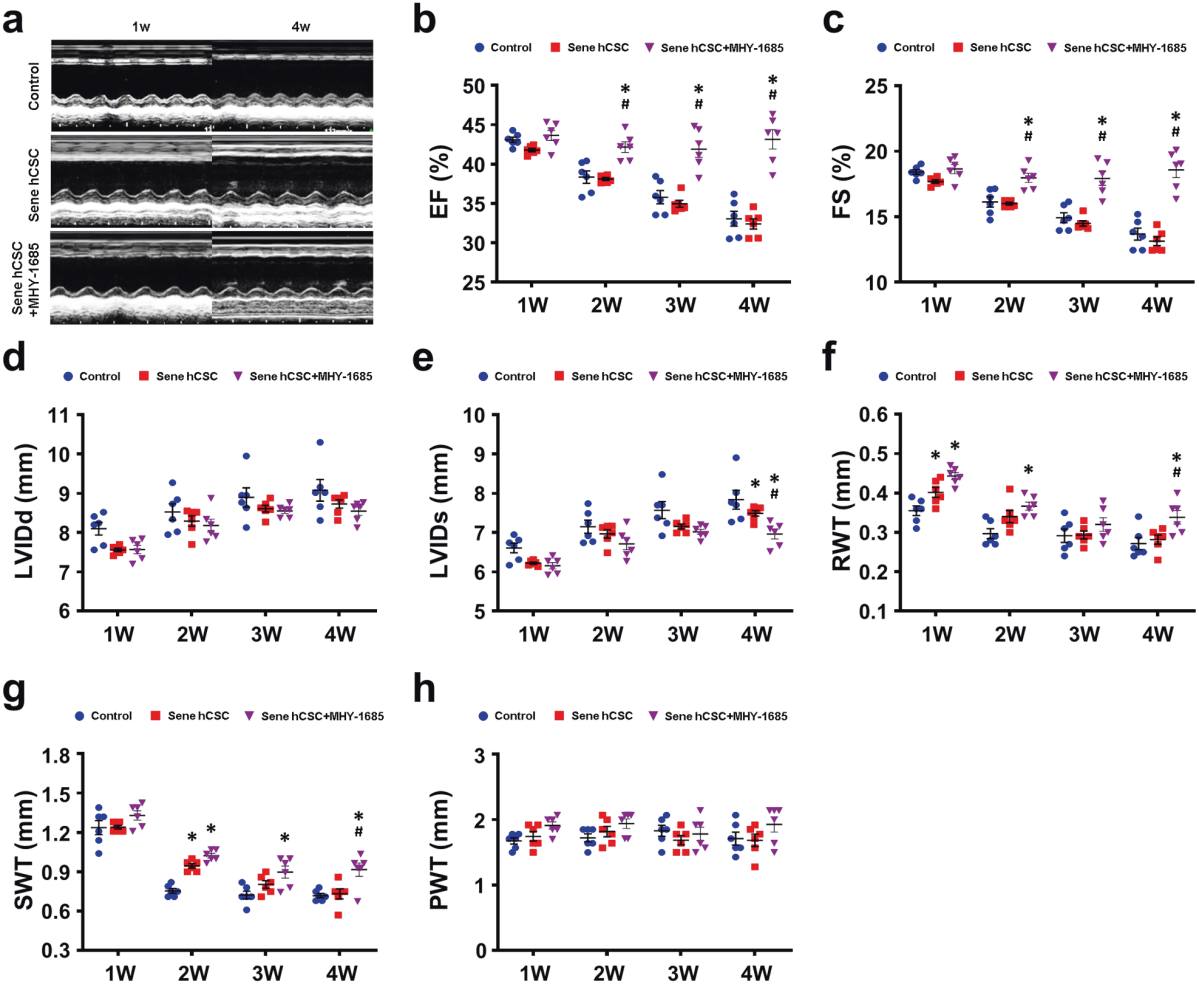
MHY-1685-primed hCSCs improve heart function while reducing adverse remodeling. **a** Representative images of the M-mode of four experimental groups at 1 and 4 weeks postintervention. **b** Left ventricular ejection fraction (EF), **c** Left fractional shortening (FS), **d** Left ventricular internal diastolic dimension (LVIDd), **e** Left ventricular internal systolic dimension (LVIDs), **f** Relative wall thickness (RWT), **g** Septal wall thickness (SWT), **h** Posterior wall thickness (PWT). n = 6. (*p < 0.05 vs. control, ^#^p < 0.05 vs. senescent hCSC). Data are shown as the mean ± S.E.M.

Subsequently, to evaluate the therapeutic efficacy of hCSCs, echocardiograms were used to measure cardiac functions on a weekly basis in the rats (Supplementary Fig. 4). Echocardiography showed that the MHY-1685-primed hCSC group exhibited significantly improved cardiac functions compared to that of the other groups (Fig. 5a), as determined by the EF (Fig. 5b) and FS (Fig. 5c). Furthermore, the parameters of cardiac remodelings, such as the LVIDd, the LVIDs, and the relative wall thickness, showed significantly reduced pathological remodeling in the MHY-1685-primed hCSC group compared to the other groups (Fig. 5d-f). The SWT, which is the wall thickness of the infarcted wall, was significantly thicker in the MHY-1685-primed hCSC group than in the other groups at the end-point (Fig. 5g). However, there was no significant difference in the PWT, which is the wall thickness of the noninfarcted wall, in all groups (Fig. 5h). Based on these results, we demonstrated that rejuvenated hCSCs primed with MHY-1685 regain therapeutic potential for cardiac restoration, which leads to improved cardiac function and reduced adverse cardiac remodeling.

### hCSC-primed MHY-1685 reduces cardiac fibrosis and protects the myocardium from ischemic injury

**Fig. 6:**
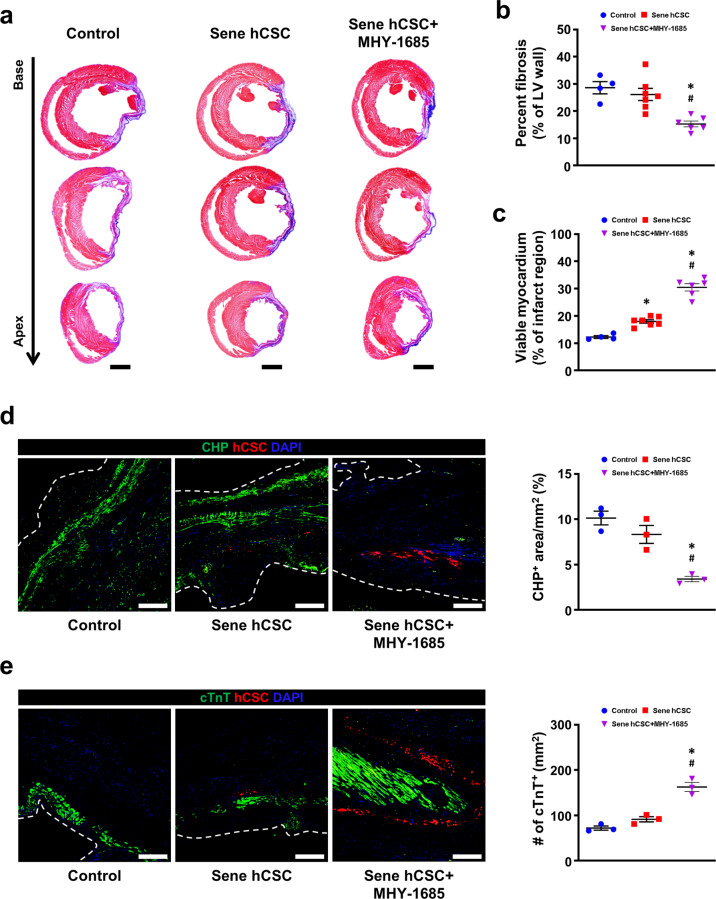
hCSC-primed MHY-1685 reduces adverse cardiac remodeling and protects the myocardium against ischemic injury. **a** Representative images of Masson’s trichrome staining using heart tissues harvested 4 weeks after intervention. Scale bar: 2000 µm. **b**, **c** Quantification summary of the percentage of fibrosis and viable myocardium. *n* = 4–7. (*p < 0.05 vs. control, ^#^*p* < 0.05 vs. senescent hCSC). **d** Representative images of denatured collagen in the infarct zone and their quantification summary. CHP (green), DiI-labeled hCSCs (red), DAPI (blue). *n* = 3. (*p < 0.05 vs. control, ^#^*p* < 0.05 vs. senescent hCSC). Scale bar: 100 µm. **e** Representative images of cardiomyocytes in the infarct zone and their quantification summary. cTnT (green), DiI-labeled hCSCs (red), DAPI (blue). *n* = 3. (*p < 0.05 vs. control, ^#^*p* < 0.05 vs. senescent hCSC). Scale bar: 100 µm. Data are shown as the mean ± S.E.M.

To investigate whether the transplantation of MHY-1685‒primed hCSCs affected cardiac fibrosis, we performed Masson’s trichrome (MT) staining at 4 weeks (Fig. 6a). The percentage of fibrotic tissues in the left ventricular wall was lower and the viable myocardium was higher in the MHY-1685-primed hCSC group than in the other groups (Fig. 6b, c). Denatured collagen continuously exacerbates adverse cardiac remodeling and fibrosis^23^, but MT staining cannot distinguish between intact collagen and denatured (or damaged) collagen, which indicates the extent of injury^24^. Therefore, we used CHP (collagen hybridizing peptide) to visualize and evaluate the deposition of denatured (or damaged) collagen and the activity of tissue remodeling in the infarcted area. The accumulation of denatured collagen was significantly reduced in the MHY-1685-primed hCSC group compared to the other groups, indicating that the extent of tissue damage and fibrosis was mitigated (Fig. 6d, Supplementary Fig. 5). As verified by MT staining, the number of surviving myocardium was higher in the MHY-1685‒primed hCSC group than in the other groups (Fig. 6e). Interestingly, hCSCs surrounded the surviving myocardium in the MHY-1685-primed hCSC group. Taken together, these findings suggest that hCSCs rejuvenated by priming MHY-1685 reduce cardiac fibrosis by inhibiting the production of denatured collagen and protecting the myocardium from ischemic injury.

### Priming MHY-1685 potentiates the differentiation potential of hCSCs into ECs in vivo

**Fig. 7:**
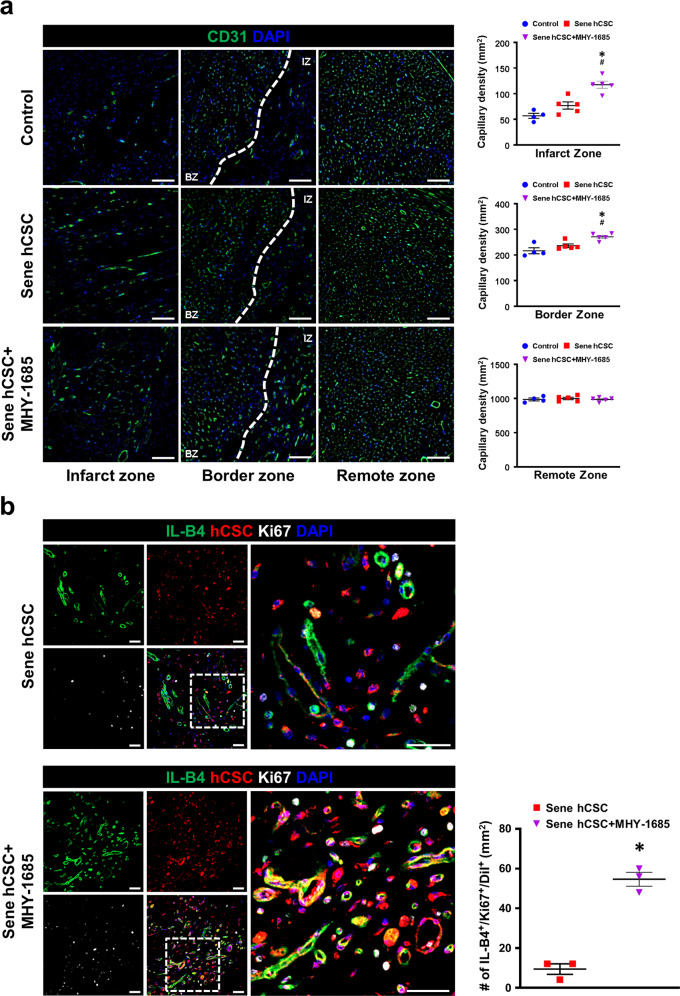
MHY-1685-primed hCSCs improve vascular regeneration through angiogenesis and differentiate into ECs. **a** Representative images of the capillary density in the infarct zone, border zone, and remote zone 4 weeks after MI and their quantification summary. CD31 (green), DAPI (blue). *n* = 4–5. (*p < 0.05 vs. control, ^#^*p* < 0.05 vs. senescent hCSC). Scale bar: 100 µm. **b** Representative images of hCSCs differentiated into ECs and proliferation in the infarct zone and quantification summary. IL-B4 (green), DiI-labeled hCSCs (red), Ki-67 (white), DAPI (blue). *n* = 3. (*p < 0.05 vs. senescent hCSC). Scale bar: 30 µm. Data are shown as the mean ± S.E.M.

Four weeks after MI, to determine whether MHY-1685‒primed hCSC transplantation enhanced vascular regeneration in infarcted hearts, capillaries were visualized in the infarct zone using CD31 and isolectin B4 (IL-B4). The results of the CD31-positive immunohistochemical staining showed that the capillary density in the MHY-1685-primed hCSC group was significantly higher than that in the other groups in the infarct zone as well as in the border zone, but there was no significant difference in the capillary density of the remote zone in all groups (Fig. 7a). Subsequently, we quantified the direct de novo vessel formation and proliferative potential of hCSCs by containing Ki-67 and IL-B4 and demonstrated that the number of IL-B4^+^/Ki-67^+^/DiI^+^ cells increased in the MHY-1685‒primed hCSC group compared to in the senescent hCSC group (Fig. 7b). In addition, we performed histological analysis to determine whether the transplanted hCSCs differentiated into CMs using cTnT as the CM-specific marker. As a result, the hCSC group did not express any cTnT-positive hCSCs, while cTnT-positive hCSCs were found in the hCSC+MHY-1685 group (Supplementary Fig. 6). However, the number of cTnT-positive hCSCs in the hCSC+MHY-1685 group was quite low and did not seem to have a rectangular shape like a mature CM shape. Therefore, it appears that transplanted hCSCs are insufficient at improving cardiac function by regenerating cardiac muscle in the infarcted area. These results indicate that hCSCs rejuvenated by priming with MHY-1685 exhibited improved potential for proliferation and differentiation into the EC lineage in infarcted hearts, leading to de novo vessel formation and cardiac repair.

## Discussion

Replicative senescence is essentially cell cycle arrest during the course of in vitro cell expansion^13^. This cellular senescence leads to reduced cellular function and therapeutic efficacy of stem cell transplantation; thus, overcoming cellular senescence is a promising strategy to enhance the therapeutic effects of stem cell-based therapies^25^. In this study, we demonstrate that modulation of autophagy via the priming of hCSCs with MHY-1685 inhibits senescence and enhances the therapeutic potential of hCSCs for cardiac repair. Our data indicate that (i) MHY-1685 improves cellular function by reducing intracellular ROS and mitochondrial superoxide production in hCSCs; (ii) MHY-1685 attenuates senescence by modulating autophagy through the inhibition of mTOR in hCSCs; (iii) MHY-1685‒primed hCSCs maintain their stemness properties, which provide the potential for differentiation into ECs and SMCs in in vitro cell cultures; (iv) transplantation of MHY-1685‒primed hCSCs significantly improves heart function while reducing adverse remodeling; and (v) MHY-1685‒primed hCSCs differentiate into ECs.

The role of mTOR signaling in cellular senescence is not clearly defined, but several studies have reported that mTOR activation via different stimuli could be involved in the induction of senescence. Astle MV et al. reported that the mTOR-dependent regulation of p53 translation and stabilization induced senescence-associated β-galactosidase activity^26^. Recently, Zhang et al. induced the downregulation of mTOR signaling by using rapamycin before the MSC senescence induced by D-galactose occurred. Treatment of MSCs with rapamycin for 24 h significantly alleviated cellular senescence, which was accompanied by reduced ROS production and the downregulation of p-Jun N-terminal kinases (JNK) and p-38^27^. Our previous study also demonstrated that chronic inhibition of mTOR by rapamycin attenuated cellular senescence and markedly improved the functions of hCSCs^14^. As shown in this study, we demonstrated that MHY-1685 also inhibited mTOR signaling in hCSCs, resulting in a higher cellular survival rate in the presence of oxidative stress, an increased proliferation rate with a short cell population doubling time, and the sustained stemness with differentiation potential. In addition, long-term priming of hCSCs with MHY-1685 significantly reduced the number of β-gal-positive cells and the expression of senescence-associated proteins through the p53/p21^CIP1^ and p16^INK4A/pRb^ tumor suppressor pathways. Taken together, these findings suggest that mTOR activation induces cellular senescence and that MHY-1685, as a novel mTOR inhibitor, attenuates the senescent phenotype of hCSCs.

Autophagy represents a crucial pathway for cellular survival and homeostasis maintenance under both physiological and stressful conditions by removing injured cytoplasmic organelles and damaged macromolecules^28^. The effects of autophagy on cellular senescence and the corresponding mechanisms have not been fully evaluated. The autophagy pathway may be activated during the transition of cells from a proliferative phase to a senescent phase. Zheng et al.^29^ reported that autophagy increased when MSCs entered a state of replicative senescence, with p53 playing an important role in increasing autophagic activity. Indeed, MSCs cultured in a medium of high glucose concentration exhibited not only early senescence, as evidenced by telomeric shortening and genomic instability, but also autophagy, as reflected by increased autophagy-related gene expression and an augmented LC3-II conversion rate. However, it is still unclear whether cells become senescent due to autophagy or despite autophagy. Ceccariglia et al. reported that the modulation of autophagy affected MSC therapeutic properties by reducing inflammation, apoptosis, and oxidative stress in cells^30^. In this study, we demonstrated that MHY-1685 enhanced the expression of autophagy-related genes in hCSCs via long-term mTOR inhibition, which prevented the cells from entering senescence and terminal differentiation programs. Beyond this anti-senescent effect, priming with MHY-1685 upregulated the expression of not only stemness markers but also angiogenic genes in hCSCs, which might have facilitated the assembly of new vascular structures in the infarcted hearts. Therefore, we believe that autophagy is a dynamic recycling system that produces new energy for cell survival and that the modulation of autophagy via mTOR inhibition may allow cells to rejuvenate themselves very efficiently.

From a cell-based therapy perspective, transplantation of hCSCs has some limitations, such as the quality and quantity of ex vivo expanded hCSCs. Modulating autophagy/senescence in hCSCs prior to transplantation is an attractive strategy for enhancing the survival and engraftment rate of hCSCs and for promoting their proangiogenic and differentiation characteristics. In this study, we provide evidence that MHY-1685, a novel mTOR inhibitor, enables the restoration of autophagy in vitro and inhibits senescence in hCSCs, thereby reinforcing the hypothesis that the intrinsic aging clock of hCSCs can be pharmacologically manipulated.

## Supplementary information

Supplementary Information
