# Author Correction: Human cardiac stem cells rejuvenated by modulating autophagy with MHY-1685 enhance the therapeutic potential for cardiac repair

**DOI:** 10.1038/s12276-022-00807-y

**Published:** 2022-07-26

**Authors:** Ji Hye Park, Hyeok Kim, Hyung Ryong Moon, Bong-Woo Park, Jae-Hyun Park, Woo-Sup Sim, Jin-Ju Kim, Hye Ji Lim, Yeon-Ju Kim, Seung Taek Ji, Woong Bi Jang, Vinoth Kumar Rethineswaran, Le Thi Hong Van, Ly Thanh Truong Giang, Jisoo Yun, Jong Seong Ha, Kiwon Ban, Hae Young Chung, Sang Hong Baek, Hun-Jun Park, Sang-Mo Kwon

**Affiliations:** 1 Laboratory for Vascular Medicine and Stem Cell Biology, Medical Research Institute, Department of Physiology, School of Medicine, Pusan National University, Yangsan, 50612 Republic of Korea; 2 R&D Center for Advanced Pharmaceuticals & Evaluation, Korea Institute of Toxicology, 141 Gajeong-ro, Yuseong-gu, Daejeon, 34114 Republic of Korea; 3 Department of Medical Life Science, College of Medicine, The Catholic University of Korea, 222 Banpo-Daero, Seocho-Gu, Seoul, 137701 Republic of Korea; 4 Laboratory of Medicinal Chemistry, College of Pharmacy, Pusan National University, Busan, 46241 Republic of Korea; 5 Department of Biomedical Sciences, City University of Hong Kong, Tat Chee Avenue, Kowloon, Hong Kong SAR; 6 Molecular Inflammation Research Center for Aging Intervention, College of Pharmacy, Pusan National University, Busan, 462414 Republic of Korea; 7 Division of Cardiology, Department of Internal Medicine, The Catholic University of Korea, 222 Banpo-Daero, Seocho-Gu, Seoul, 137701 Republic of Korea

**Keywords:** Heart stem cells, Stem-cell therapies

Correction to: *Experimental & Molecular Medicine* 10.1038/s12276-021-00676-x, published online 28 September 2021

The color of the histogram in Figure 1.h and Figure 2.b were incorrectly inserted; the figures should have appeared as shown below.
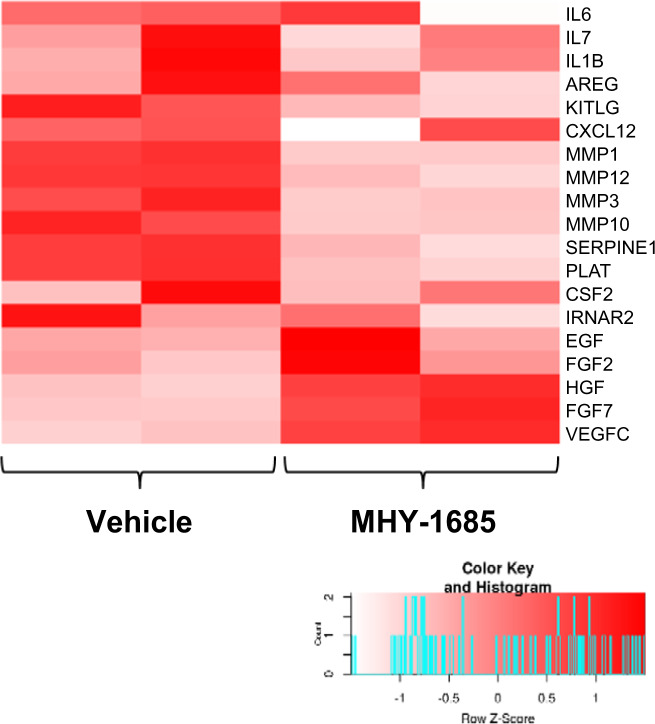

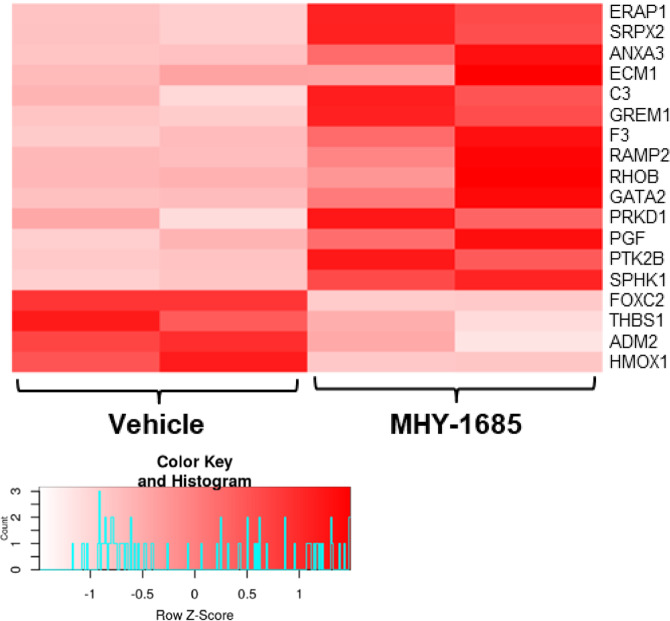


The original article has been corrected.

