# Supplementary material for: Human cardiac stem cells rejuvenated by modulating autophagy with MHY-1685 enhance the therapeutic potential for cardiac repair
Source: Exp Mol Med. 2021 Sep 28;53(9):1423–36. doi: 10.1038/s12276-021-00676-x (PMC8492872; doi:10.1038/s12276-021-00676-x)

Human cardiac stem cells rejuvenated by modulating autophagy with MHY-1685 enhance the therapeutic potential for cardiac repair

Ji Hye Park[1,2,*] , Hyeok Kim[3,*], Hyung Ryong Moon[4,*], Bong-Woo Park[3], Jae-Hyun Park[3], Woo-Sup Sim[3], Jin-Ju kim[3], Hye Ji Lim[1], Yeon-Ju Kim[1], Seung Taek Ji[1], Woong Bi Jang[1], Vinoth Kumar Rethineswaran[1], Le Thi Hong Van[1], Ly Thanh Truong Giang[1], Jisoo Yun[1], Jong Seong Ha[1], Kiwon Ban[5], Hae Young Chung[6], Sang Hong Baek[7,†], Hun-Jun Park[3,7,†], Sang-Mo Kwon[1,†]

[1]Laboratory for Vascular Medicine and Stem Cell Biology, Medical Research Institute, Department of Physiology, School of Medicine, Pusan National University, Yangsan 50612, Republic of Korea

[2]R&D Center for Advanced Pharmaceuticals & Evaluation, Korea Institute of Toxicology, Republic of Korea

[3]Department of Medical Life Science, College of Medicine, The Catholic University of Korea, 222 Banpo-daero, Seocho-gu, Seoul, 137701, Republic of Korea

[4]Laboratory of Medicinal Chemistry, College of Pharmacy, Pusan National University, Busan 46241, Republic of Korea

[5]Department of Biomedical Sciences, City University of Hong Kong, Tat Chee Avenue, Kowloon, Hong Kong SAR

[6]Molecular Inflammation Research Center for Aging Intervention, College of Pharmacy, Pusan National University, Busan 462414, Republic of Korea

[7]Division of Cardiology, Department of Internal Medicine, The Catholic University of Korea, 222 Banpo-daero, Seocho-gu, Seoul, 137701, Republic of Korea

* These authors contributed equally.

[†] Correspondence to: Sang-Mo Kwon: smkwon323@hotmail.com; Hun-Jun Park: cardioman@catholic.ac.kr; Sang Hong Baek: whitesh@catholic.ac.kr

Supplementary Figure

Supplementary Figure 1. Cytoprotective effect of MHY-1685 against H2O2-induced oxidative stress. a chemical structure of MHY-1685 **b** Screening of new anti-senescence candidate. Senescent hCSCs were treated MHY-1681~5 for 24h and then protein was extracted by RIPA. The relative expression of the anti-senescence marker SMP30 was examined by western blot assay. **c** Cytotoxicity assay of MHY-1685 in hCSCs. hCSCs were cultured in 96-well plates and treated with various of concentration MHY-1685 for 24h. n= 4. (*p< 0.05 vs control). **d** Mitochondrial superoxide production as measure by MitoSOX staining in 0-10 μM treated hCSCs. hCSCs were incubated with MHY-1685 for 24h prior to the addition of 600μM H2O2 for 1h. n= 3. (*p< 0.05 vs -H2O2, $^{\#}p <$ 0.05 vs +H2O2 and 0 μM). **e** Cellular ROS production was measured by H2-DFFDA assay. hCSCs were treated MHY-1685 for 24h prior to the addition of 600μM H2O2 for 1h. n=3 (*p< 0.05 vs -H2O2 and -MHY-1685, $^{\#}p <$ 0.05 vs +H2O2 and -MHY-1685). **f** Cell death assay as measured by Annexin V/7-AAD. (*p< 0.05 vs -H2O2, $^{\#}p$ < 0.05 vs 0 μM). Data are shown as the mean ± S.E.M.

Supplementary Figure 2. MHY-1685 primed hCSCs didn't show oncogenic phenotype. Matrigel mixed single-cell suspension were mixed with PBS (Vehicle), $1x10^7$ hCSCs, MHY-1685 treated hCSC or cancer cell line (DLD-1, HCT-8) and injected subcutaneously in the left and right flank region of BALB/c SCID immune-deficient mice (n=5). The size of tumors was measured by caliper. After 2weeks, mice were sacrificed and tumors were excised. Graph shows tumor volume [$V = r_w^2 \times r_L) / 2$].

Supplementary Figure 3. The differentiation capacity of senescent hCSCs into myocardium. a Enrichment of muscle development/proliferation related GO terms of genes that were significantly regulated in MHY-1685–primed hCSCs. **b** Cardiomyogenic differentiation potential as measured by immunocytochemistry using the α-SA. α-SA (green), DAPI (blue). Scale bar: 100 μm.

Supplementary Figure 4. Schematics outlining the *in-vivo* experimental strategy and schedule of transplantation of hCSCs after MI modeling.

Supplementary Figure 5. hCSCs primed MHY-1685 reduce cardiac fibrosis and denatured collagen. Representative images of CHP staining (scale bar: 100 μm) and MT staining (scale bar: 200 μm) in infarcted area. CHP (green), DiI-labeled hCSCs (red), DAPI (blue).

Supplementary Figure 6. The differential potential of hCSCs into cardiomyocytes. Representative images of

hCSCs differentiated into CMs in the infarct zone. cTnT (green), DiI-labeled hCSCs (red), DAPI (blue). Scale bar: 40 µm (Left), 20 µm (Right)

Supplementary information accompanies the manuscript on the Experimental & Molecular Medicine` website (http://www.nature.com/emm/)

Supplementary Figure 1.

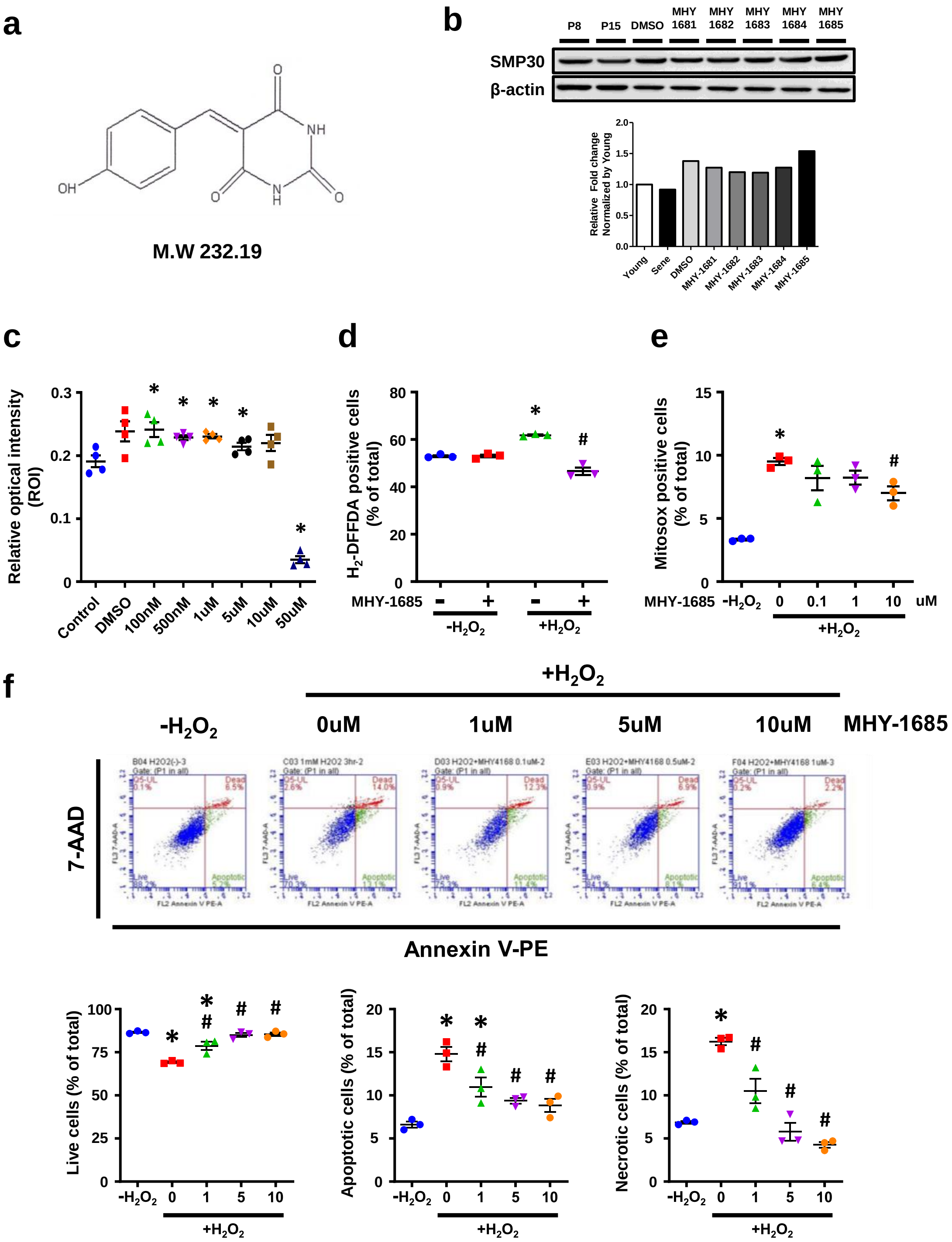

Supplementary Figure 2.

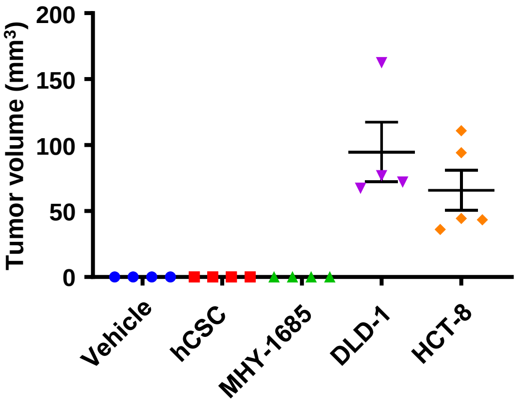

Supplementary Figure 3.

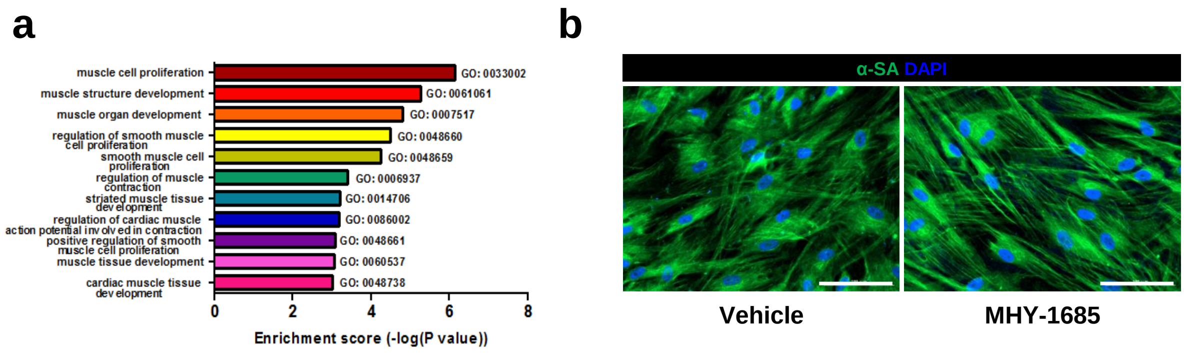

Supplementary Figure 4.

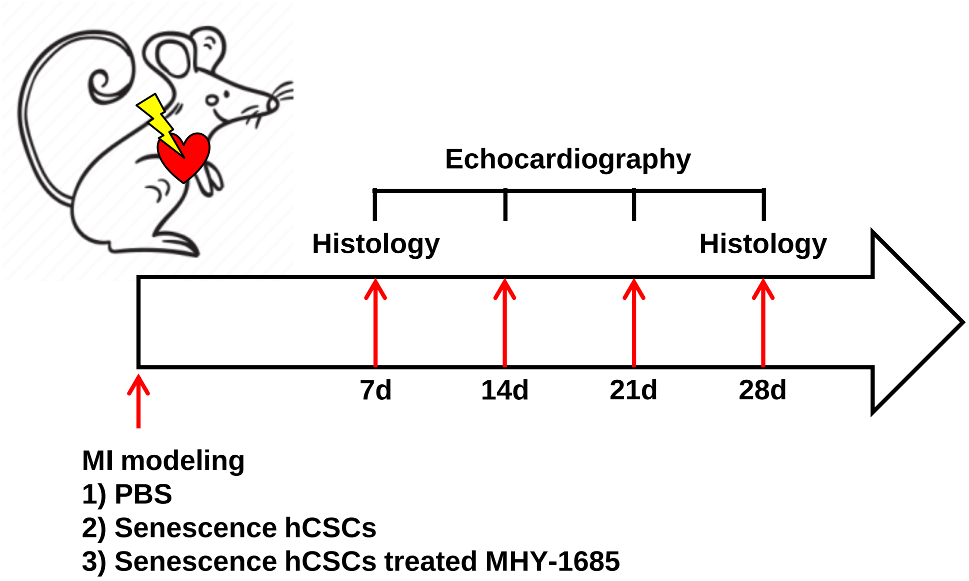

66 **Supplementary Figure 5.**

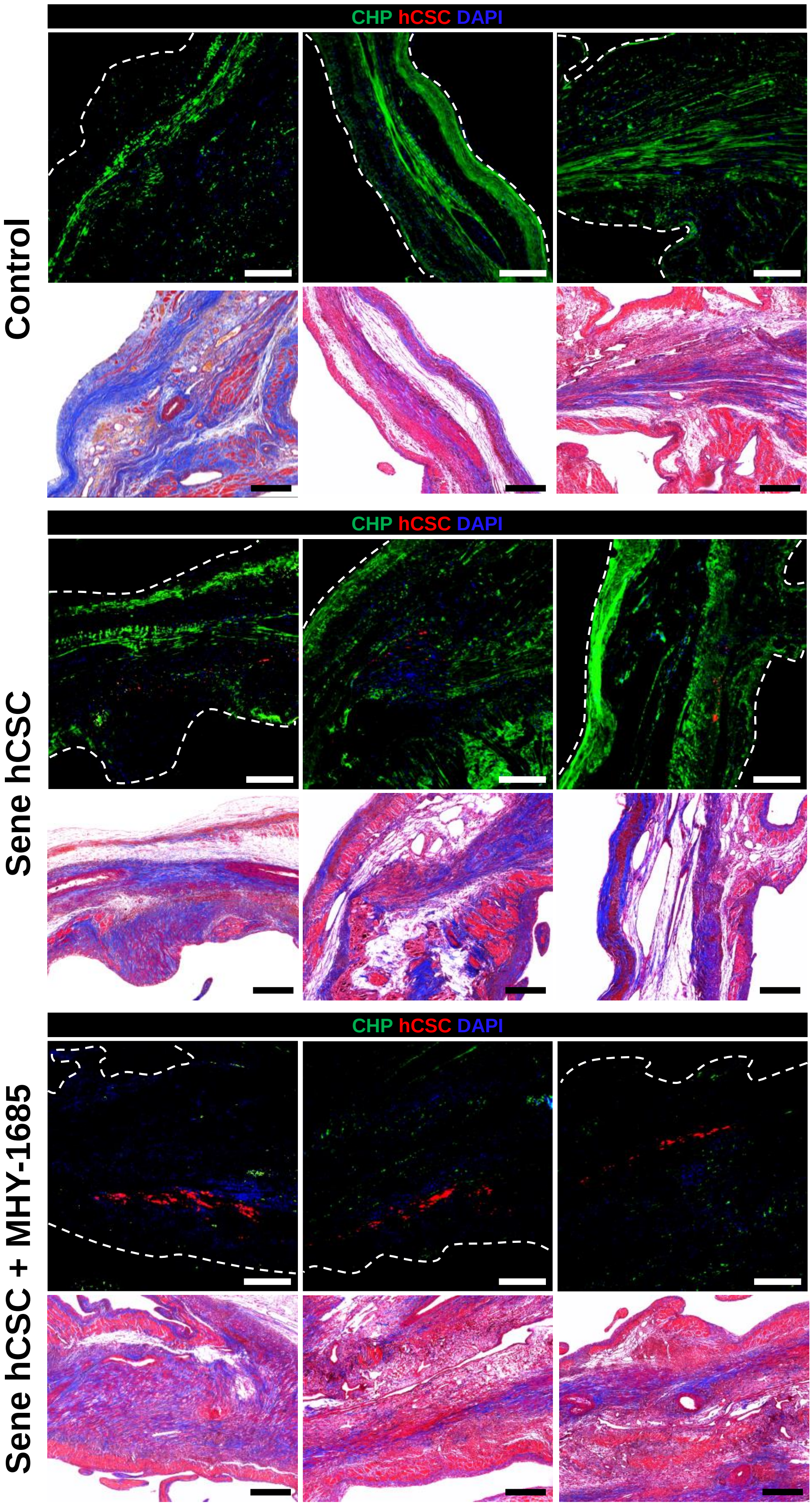

67

Supplementary Figure 6.

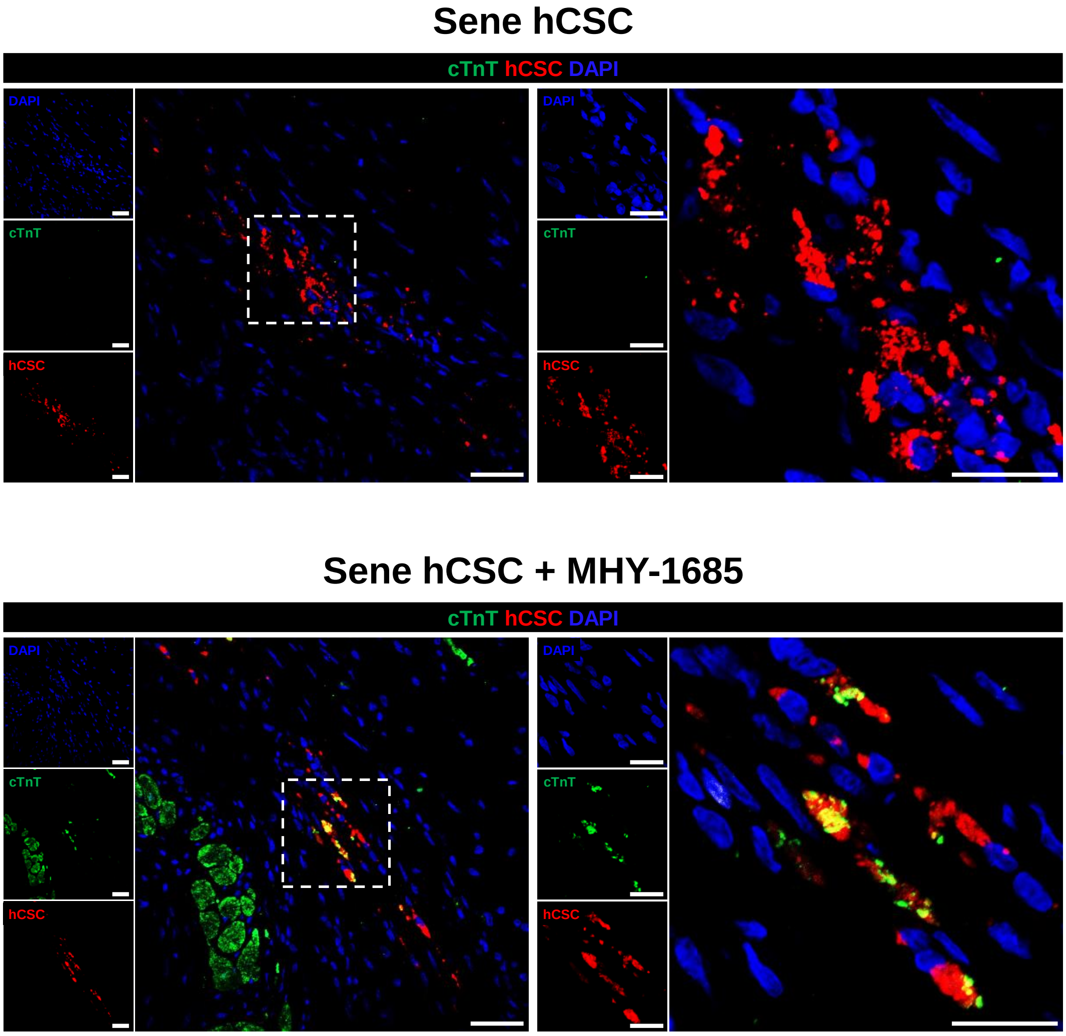

Supplement: Supplementary file 1 — Supplementary Information [file 12276_2021_676_MOESM1_ESM.pdf]
